# Intersection of gender and class in the assessment of self-rated health in the Spanish population: a classification tree study

**DOI:** 10.1186/s12939-026-02897-w

**Published:** 2026-05-29

**Authors:** Jordi Gumà-Lao, Núria Pedrós Barnils, Daniel La Parra-Casado

**Affiliations:** 1 Centre for Demographic Studies, Barcelona, Spain; 2 Institute for Public Health and Nursing Research, University of Bremen, Bremen, Germany; 3 Department of Epidemiology and Global Health, Umeå University, Umeå, Sweden; 4 Department of Sociology II, University of Alicante, Alicante, Spain

**Keywords:** Health inequity, Self-rated health, Machine learning, Intersectional framework

## Abstract

**Aim:**

This study assesses the influence of various specific health conditions as activity limitations and groups of chronic diseases, in estimating poor self-rated health for the Spanish population aged 30–60, stratified by the intersections of gender and social class. Moving beyond unidimensional social gradients, the research utilises decision tree analysis to identify specific configurations of SRH responses that align with the epidemiological profiles of distinct intersectional strata.

**Methods:**

Data were drawn from the 2023 Spanish Health Survey (ESdE), focusing on a sample of 10,210 adults aged 30–60. This age bracket was selected to capture health inequality dynamics during the working life-course. Specific machine learning decision trees (J48 algorithm) were employed to assess the pathways for classifying individuals with poor SRH across six distinct intersectional groups.

**Results:**

Resulting decision trees differ significantly in composition, shape, and length across strata. A clear trend emerged: less skilled groups exhibited higher tree complexity, reflecting a more rapid accumulation of chronic illnesses at earlier ages.

**Conclusion:**

This research contributes to the emerging field of study regarding what truly underlies individuals’ general assessment of their health. The resulting decision trees illustrate the social gradient in health in a novel way that enriches the understanding of health inequalities. Instead of simply showing a linear trend across a one-dimensional variable, these trees reveal that as disadvantages accumulate through the intersection between gender and social class, the combination of relevant diseases do not only become more complex, but also distinct, in specific and unique ways for each group, as the intersectional framework predicts.

**Supplementary Information:**

The online version contains supplementary material available at 10.1186/s12939-026-02897-w.

## Introduction

Self-rated health (SRH) is a widely used measure in diverse survey contexts, as it provides a single subjective indicator that has been consistently associated with mortality, healthcare utilization, and various health outcomes [1]. This health indicator, understood as an individual’s self-assessment of their overall health status, is not merely an individual construct. Rather, it represents a multidimensional process shaped and reproduced through interpersonal and social interactions [2]. This perspective aligns with the psychological theory of intersubjectivity, which posits that differences in subjective perceptions emerge primarily from social contexts rather than individual attributes [3]. Among individuals with similar sociodemographic profiles, understandings of health tend to converge, a dynamic explained by social comparison processes [4]. Such comparisons may help clarify why older adults often evaluate their health more positively: the normalization of age-related limitations can lead to more favourable self-assessments [5].

Health inequity literature describes a gradient in health: those with better social positions within social structures are more likely to have better health status. Disadvantaged groups, particularly women with lower educational levels, tend to exhibit less favourable objective health profiles than their more advantaged counterparts [6]. Paradoxically, a “reporting bias” has been described: even when facing similar objective health challenges, disadvantaged individuals are less likely to categorise their health as “poor” than their more advantaged counterparts, despite their higher risks of early-onset chronic diseases, functional disabilities at younger ages, and reduced life expectancy [7 –9].

Our analysis focuses on adults aged 30 to 60. This choice responds to the fact that in previous research the relationship between objective health and self-rated health have focused on older populations, who typically exhibit greater heterogeneity in health status [10 , 11]. Since health inequalities emerge through processes unfolding across the life course, examining these dynamics among individuals in their working ages may enhance our understanding of inequity patterns and help inform policies aimed at mitigating them.

The intersectionality approach provides a framework for describing and interpreting health inequities that integrates multiple systems of oppression such as racism, sexism, classism, ageism, and heteronormativity. Originally promoted by Black feminism in the United States, it was later adopted by academia to study the intersections of gender and race [12 , 13]. It has progressively been transferred to other social contexts to analyse the interdependence among diverse forms of power relations [14 , 15]. Adopting an intersectional framework to understand how differently intersectionally defined individuals experience and perceive their own health status may be valuable for a variety of reasons. First, every social stratum may be exposed to specific health determinants and, as a consequence, present differences in the health problems they encounter. For example, individuals working in manual occupations may experience different types of health problems than those employed in non-manual jobs [16]. Likewise, the specific ways in which sexism is expressed, whether through interpersonal interactions or through the living conditions it produces, vary according to socioeconomic position [17]. Second, both the accumulation of disadvantage and the specific forms these disadvantages take may lead individuals to experience poorer health, reflected in a higher burden of disease or multimorbidity, as well as increased severity of the conditions they face, in line with the syndemic hypothesis [18]. Finally, because an intersectional position entails a shared experience of social structures and discrimination, this situatedness, which refers to the way an individual’s specific social and historical location shapes their perspective, influences how an individual perceive and judge their own health [19]. Consequently, individuals within the same intersectional position in the social structure often assess their health more similarly than those in different social locations. Concretely, we explore the intersections between gender and occupational social class. Gender is understood as a system of social relations that organises roles, resources, and power relations hierarchically between men and women, while occupational social class is presented as a position within labour market hierarchies that reflects differentiated exposure to material conditions, working environments, and life chances.

Given that intersectionality emphasizes that health inequalities are produced through the interaction of multiple axes of stratification, which may generate distinct configurations of health problems and lived experiences, an analytical strategy is needed that can identify heterogeneous and non-additive patterns across intersectionally defined subgroups [20]. Decision trees are non-parametric, data-driven modelling techniques that recursively partition a sample into increasingly homogeneous subgroups according to combinations of predictor variables [21]. Unlike traditional statistical methods, which impose certain assumptions (e.g. linearity and additivity) and require to specify interaction terms in advance, decision trees identify relevant combinations of conditions in a data-driven manner without presupposing the structure of the relationship between variables. This is a substantive methodological advantage for intersectionality-informed research: this analysis remains open to the possibility that the health conditions associated with poor SRH differ not only in prevalence but in kind across intersectional strata. In the present case, they allow identifying which specific health problems, as well as which sequences or combinations of conditions, are more predictive of poor SRH. However, given the cross-sectional nature of our data, the findings of this research present a classification of a poor SRH state rather than a prediction. This approach is particularly suitable for intersectionality-informed research, as it does not impose linearity assumptions or additive effects, but instead captures complex interactions and conditional relationships in an interpretable manner [22 , 23]. Furthermore, decision trees provide a visual representation of the hierarchy of objective health conditions and how they translate into subjective health evaluations across intersectional groups. This is especially useful when comparing heterogeneous intersectional strata that may differ in both the prevalence and the salience of specific health problems.

The aim of this analysis is to examine how different health problems contribute to the construction of SRH in subgroups defined intersectionally by gender and occupational social class, using decision trees to identify differential patterns. In line with the considerations outlined above, each intersectional group is expected to exhibit specific patterns regarding which health conditions are included in the decision trees. Likewise, the frequency and combination of these conditions are expected to produce distinct trees. We hypothesize that when the relationship between health problems and SRH is clear and relatively homogeneous within an intersectional group, the decision trees will tend to be simpler and more compact. Conversely, in groups with a higher prevalence and greater diversity of health problems, the model will require more divisions to capture the heterogeneity of patterns, yielding larger and more branching trees. To sum up, strata with better health status are expected to have more concise trees.

## Data and methods

The present study analyses data sourced from the 2023 edition of the Spanish Health Survey (ESdE). The ESdE is a periodic cross-sectional survey representative of the general non-institutionalized population in Spain. This survey collects information on the health of the Spanish population in a harmonized manner within the European context. The total sample consists of 24,673 interviews, 21,032 adults (aged 15 and over), and 3,641 minors (aged 0–14). In our research, the working sample comprised individuals aged 30–60, that is, a total of 10,210 individuals. Missing values (1.98% of individuals) were treated as completely at random, yielding a final analytical sample of 10,011 individuals. The age range was set to consider the target population after leaving their parental home (the average age in Spain is 30.4 [24] and before retirement (the average effective age in Spain is 61.8 for women and 62 for men [25] due to our goal of focusing on potential differences between population within the active age range.

The health indicators selected (comprising various chronic diseases alongside two measures of disability, namely instrumental activities of daily living -IADL- and activities of daily living -ADL-) to explore their relationship with SRH are based on their prior association with this indicator. The decision to focus on chronic diseases stems from our use of the Disablement Process Model [26]. This model proposes a sequence of health deterioration leading to disablement, which begins with the diagnosis of chronic pathologies. This is key, given that the study population (aged 30–60) exhibits a health profile in which problems are incipient in most cases. Finally, the results are specific to the Spanish population and thus cannot be generalized until complemented by studies in other countries. The influence of chronic diseases on SRH has been shown to be different for women and men [27]. This is why the different chronic diseases have been grouped according to the ICD-10 chapters in order to distinguish their influence on women and men separately. As for the IADL and the ADL, both have been shown to influence SRH [28]. Furthermore, ADL is associated with a higher level of dependency, so by including both, we consider not only the fact of presenting a certain limitation but also the severity of this limitation [29].

The health indicators were operationalized as follows:

Chronic diseases. The ESdE questionnaire includes questions about 34 chronic diseases and health conditions. In our research, we focused on those chronic diseases with a medical diagnosis, which were grouped as follows: Circulatory System Diseases (High blood pressure -hypertension; Myocardial infarction; Angina pectoris / Coronary artery disease; Varicose veins in the legs; Stroke; Other heart diseases); Respiratory System Diseases (Chronic allergy; Asthma; Chronic bronchitis / emphysema / COPD); Musculoskeletal System and Connective Tissue Diseases (Osteoarthritis; Chronic neck pain; Chronic lower back pain; Osteoporosis; Permanent injuries or defects due to an accident); Endocrine, Nutritional, and Metabolic Disorders (Diabetes; High cholesterol; Thyroid problems); Mental Disorders (Depression; Chronic anxiety; Other mental problems); Neurological Disorders (Alzheimer’s and other dementias; Migraine); Digestive System Diseases (Stomach or duodenal ulcer; Chronic constipation; Hemorrhoids; Cirrhosis / Liver dysfunction); Genitourinary System Diseases (Urinary incontinence; Kidney problems; Prostate problems; Menopausal period problems); Neoplasms (Malignant tumours); Other (Chronic skin problems; Cataracts).

Limitations: Considering the age range of our working sample, the prevalence of the two common types of limitations is remarkably low (ADL − 1.4%- and IADL − 3.7%-). This fact compromises the potential results of the study. It was therefore decided to jointly consider the questions related to both the ADL (“Of the activities we are going to show you, do you usually have difficulty doing them without assistance?“, where the activities were: dressing; eating; using the toilet; bathing and showering; getting in and out of bed) and the IADL (“Do you usually have difficulty doing any of these activities by yourself and without assistance?”: Preparing meals; Using the telephone; Shopping; Taking medication, including remembering the dosage and when to take it; Performing light household tasks such as doing the laundry, making the bed, cleaning the house, etc.; Occasionally performing household tasks that require considerable effort, such as moving furniture, cleaning windows, carrying shopping, etc.; Managing their own money). The final variable was dichotomized as having no limitations and having activity limitations (i.e., difficulties performing one or more of the activities).

SRH: The variable was based on responses to the question ‘In the last twelve months, would you say your health has been very good, good, fair, poor, or very poor?”. Following common practice, the five possible answers were grouped into two categories: excellent, very good or good health (good health), and fair or poor health (poor health) [30].

Social class: The ESdE provides six different groups representative of the socioeconomic class of the household based on the occupation of the reference person within the household. These were grouped into three different groups: non-manual or managerial-professional group (directors and managers of establishments with 10 or more employees and professionals traditionally associated with university degrees; directors and managers of establishments with fewer than 10 employees and professionals traditionally associated with university diplomas and other technical support professionals; athletes and artists), intermediate (intermediate occupations and self-employed workers; supervisors and workers in skilled technical occupations), and manual workers (skilled workers in the primary sector and other semi-skilled workers; unskilled workers).

Six intersectional strata were created by combining the three categories from social class with two from gender to consider the intersection between socioeconomic and gender inequalities: women-manual, women-intermediate, women-professionals, men-manual, men-intermediate, and men-professionals. The absence of variables for self-identifying ethnic or racial groups prevented the enrichment of the analysis with this key social categorization. Specific machine learning decision trees for each intersectional group were employed to assess which comorbidities and their combinations define the pathway to classify individuals with poor SRH.

The J48 decision tree algorithm, an updated version of the C4.5 algorithm implemented in Weka [31], was used. This decision tree uses the information gain ratio (i.e. a form of entropy) to generate the splitting criteria for growing the tree. In that way, J48 generate groups that are the most homogeneous among themselves and heterogeneous between each other. The final tree is chosen through error-based post-pruning methods to reduce overfitting by pruning branches of the tree that increase the overall error [32].

Specifically, the J48 algorithm was implemented in a machine learning approach. That is, a k-fold validation process, with 10 folds, was used to prevent overly optimistic results and calculate an average performance. All the resulting classification trees were pruned by fixing a minimum number of cases per leaf (*N* = 25) and a confidence factor (CF) of 0.25. The first parameter was chosen based on an approximation to the principle of parsimony [33], selecting the most parsimonious model that maintained the highest classification accuracy across most intersectional groups. Additional File 1 illustrates the accuracy of each tree at different levels of the minimum number of cases per leaf. The CF is the parameter that, within the J4.8 decision tree pruning algorithm, determines the level of pessimism used to estimate the upper bound of the true error for a branch, thereby controlling the aggressiveness with which the tree is simplified. Its values range from 0 to 1, where values close to 0 reduce the risk of overfitting and improve generalisation ability on new data [34].

A machine learning approach was chosen over an exploratory approach to control for overfitting, given trees’ strong tendency to overfit the dataset on which they are trained. Furthermore, by cross-validating the dataset, the leaves of the final trees (i.e., the combinations of chronic diseases) are, under machine learning terms, predictive of poor SRH. In this cross-sectional context, the trees do not predict future health states but identify, through classification, which pathways and combinations of health conditions are associated with current poor SRH within each intersectional stratum. In other words, we ought to say for each intersectional group, which combination of chronic diseases is more predictive of poor SRH. In the field of public health, several authors have applied this specific approach for SRH [35], generalized anxiety during COVID-19 [36], and COVID-19 mortality [37].

Finally, to ensure robustness and internal validation of the results across the different trees, a random forest was performed for each stratified dataset. In essence, this ensemble algorithm highlights the importance of each included variable by constructing a large number of decision trees, thereby achieving higher classification accuracy but at the expense of lower interpretability [38]. Building an ensemble of decision trees has been shown to minimize classification errors by leveraging the predictive performance of multiple individual decision trees [39]. Consequently, random forests were used to validate the accuracy of our decision trees.

## Results

Table 1 displays descriptive characteristics of the study sample by intersectional strata. In general terms, the results confirm the expected overall health pattern: women exhibit poorer health outcomes than men. Furthermore, a social class gradient is observed, with health profiles improving as social class increases, regardless of gender. This social class gradient shows a few exceptions for some chronic diseases (though the differences are minor), including the respiratory, endocrine, mental, and neurological systems (only in men) and the genitourinary system (only in women).

**Table 1:** Sample profile of individuals aged between 30 and 60 years old according to gender and social class. Spain 2023

		Women (*N* = 5144)			Men (*N* = 4867)		
		Manual (*N* = 2258)	Intermediate (*N* = 1633)	Professional (*N* = 1253)	Manual (*N* = 2158)	Intermediate (*N* = 1679)	Professional (*N* = 1030)
Age	Average	46.2	46.1	45.6	46.6	46.7	45.7
SRH	Poor	32.71	28.17	22.43	24.5	22.04	17.09
	Good	67.29	71.83	77.57	75.5	77.96	82.91
	Total	100	100	100	100	100	100
Cardiovascular	Diagnosed	27.12	22.96	20.35	23.15	22.51	20.49
	Not diagnosed	72.88	77.04	79.65	76.85	77.49	79.51
	Total	100	100	100	100	100	100
Respiratory System	Diagnosed	21.57	25.29	23.94	15.57	19.24	22.43
	Not diagnosed	78.43	74.71	76.06	84.43	80.76	77.57
	Total	100	100	100	100	100	100
Musculoskeletal System	Diagnosed	29.41	29.52	21.79	21.41	20.96	16.21
	Not diagnosed	70.59	70.48	78.21	78.59	79.04	83.79
	Total	100	100	100	100	100	100
Endocrine	Diagnosed	25.86	27.56	26.1	21.13	23.23	22.43
	Not diagnosed	74.14	72.44	73.9	78.87	76.77	77.57
	Total	100	100	100	100	100	100
Mental	Diagnosed	19.00	19.53	15.64	10.89	9.47	9.51
	Not diagnosed	81.00	80.47	84.36	89.11	90.53	90.49
	Total	100	100	100	100	100	100
Neurological	Diagnosed	16.87	16.53	15.00	5.14	6.73	7.28
	Not diagnosed	83.13	83.47	85.00	94.86	93.27	92.72
	Total	100	100	100	100	100	100
Digestive System	Diagnosed	13.2	13.84	15.72	10.15	10.54	9.81
	Not diagnosed	86.8	86.16	84.28	89.85	89.46	90.19
	Total	100	100	100	100	100	100
Genitourinary	Diagnosed	13.46	15.62	15.48	5.56	5.96	4.85
	Not diagnosed	86.54	84.38	84.52	94.44	94.04	95.15
	Total	100	100	100	100	100	100
Tumours	Diagnosed	3.28	4.72	3.03	2.09	1.79	1.26
	Not diagnosed	96.72	95.28	96.97	97.91	98.21	98.74
	Total	100	100	100	100	100	100
Others	Diagnosed	7.84	10.47	9.82	6.67	6.67	10
	Not diagnosed	92.16	89.53	90.18	93.33	93.33	90
	Total	100	100	100	100	100	100
Limitations	Any limitation	5.00	3.98	3.11	4.31	3.39	1.46
	No limitations	95.00	96.02	96.89	95.69	96.61	98.54
	Total	100	100	100	100	100	100

Source: Spanish Health Survey (ESdE)

**Fig. 1:**
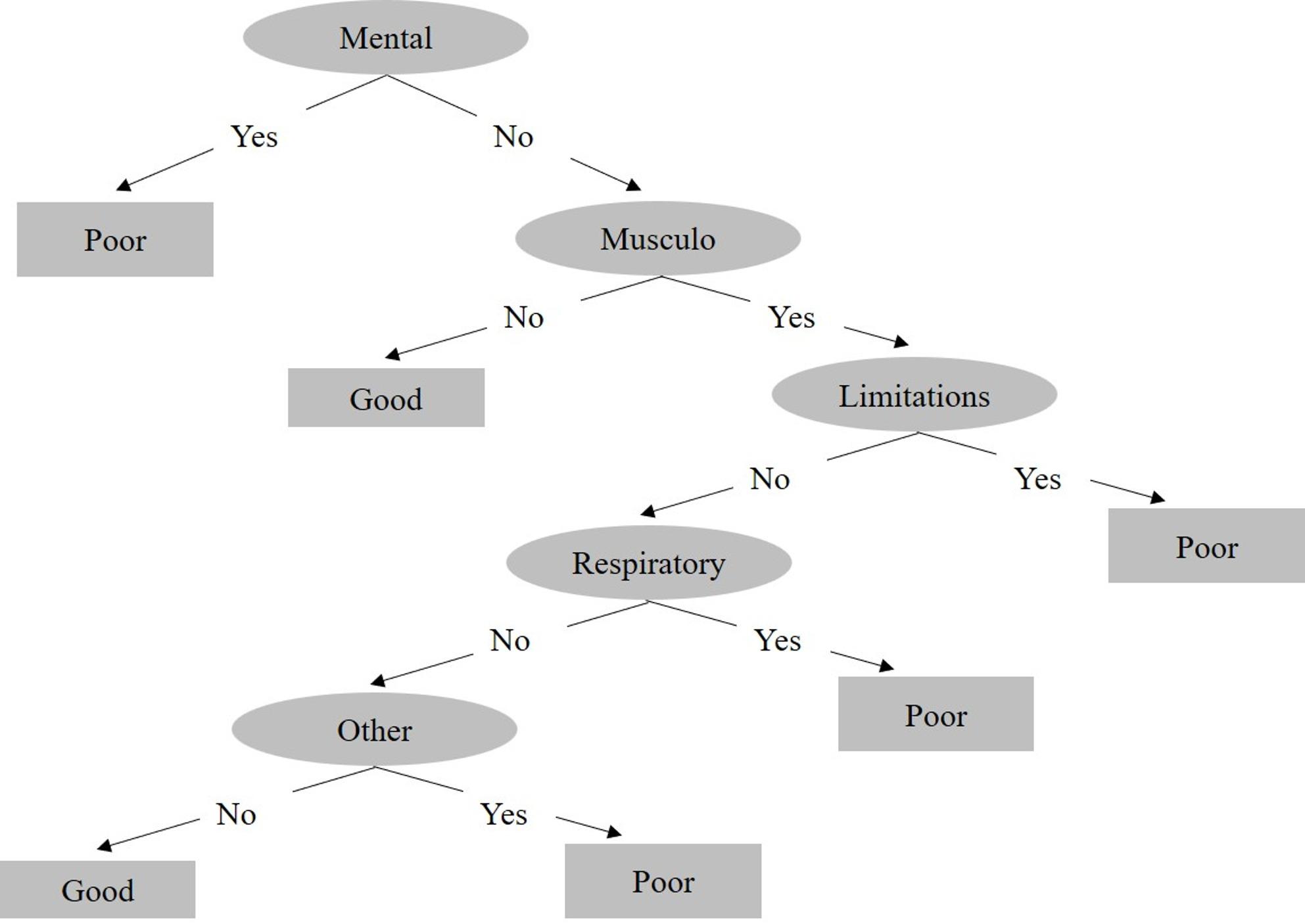
Decision tree for the classification of self-rated health for women in the manual social class group

**Fig. 2:**
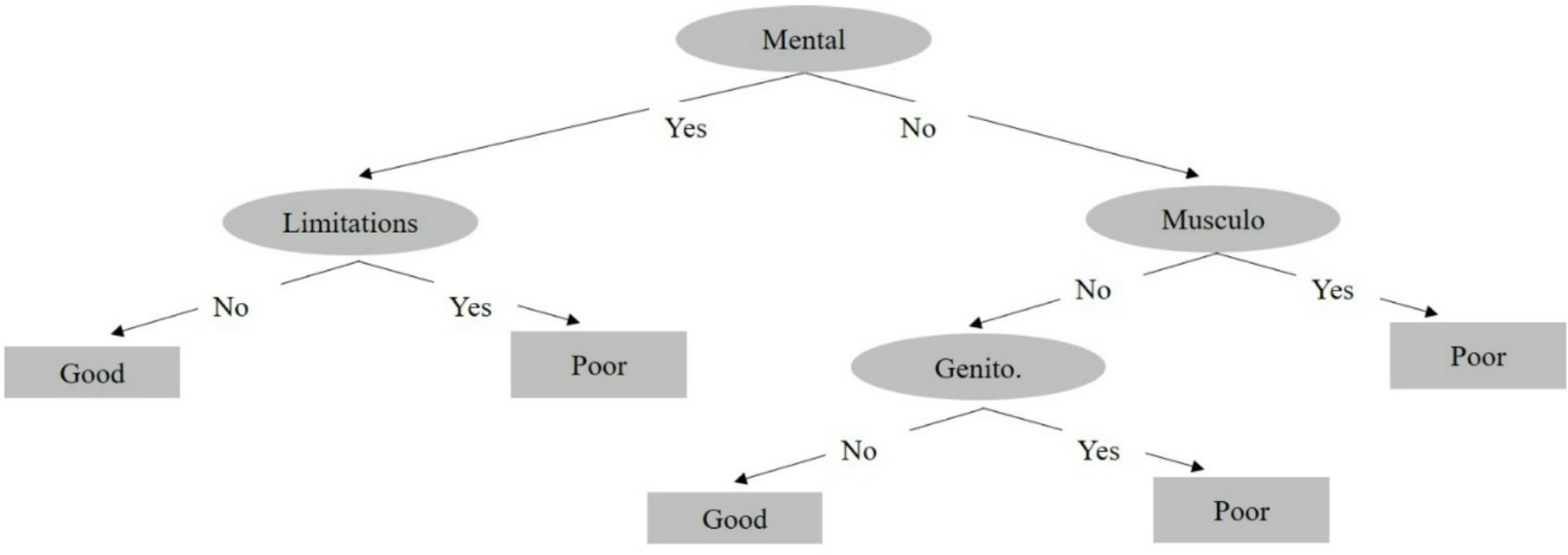
Decision tree for the classification of poor self-rated health for women in the intermediate social class group

**Fig. 3:**
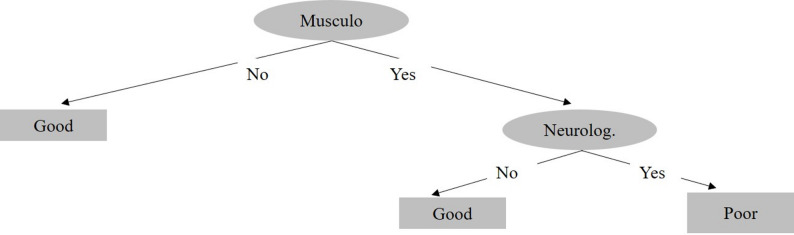
Decision tree for the classification of self-rated health for women in the professional class group

**Fig. 4:**
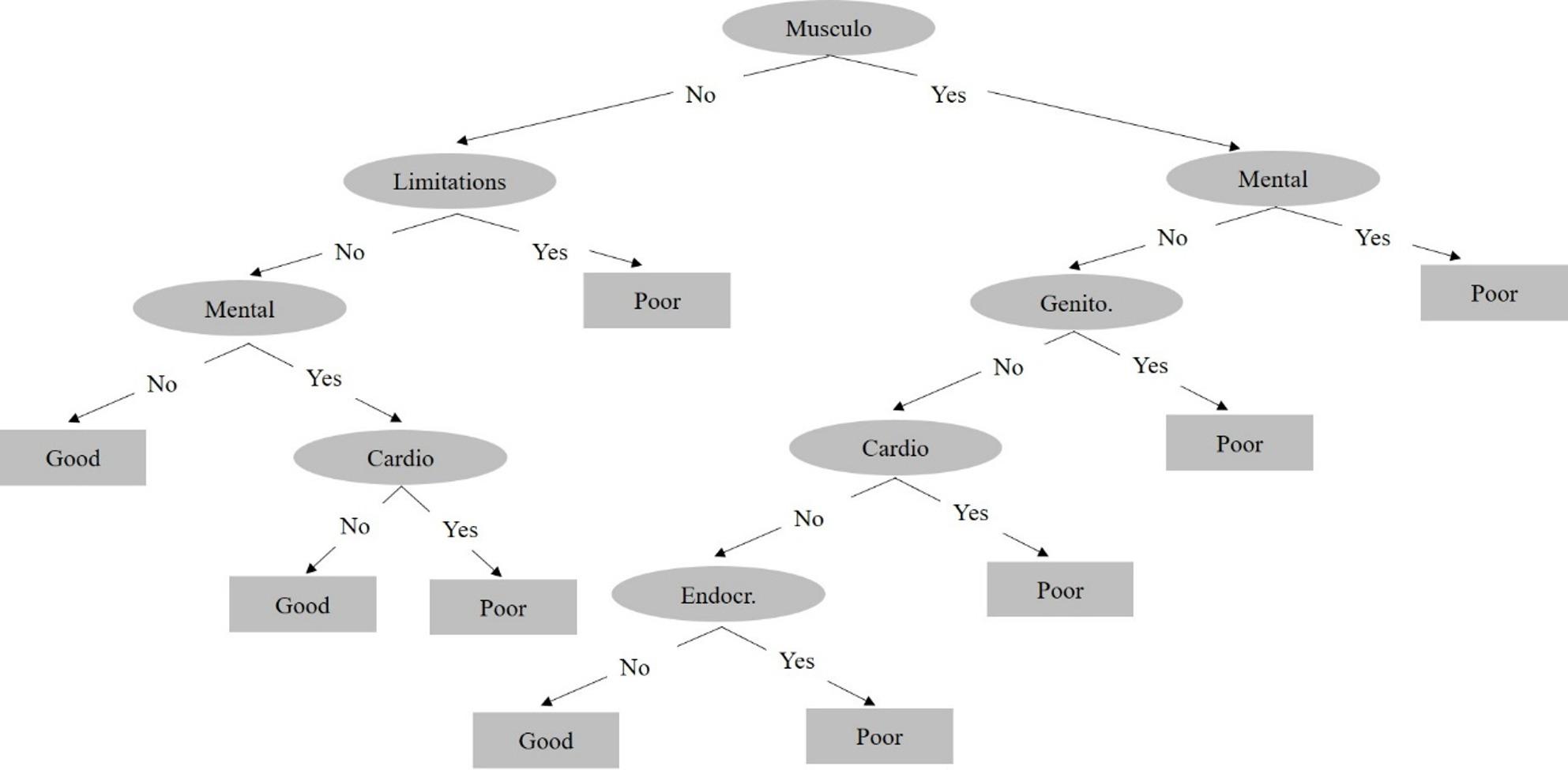
Decision tree for the classification of poor self-rated health for men in the manual social class group

**Fig. 5:**
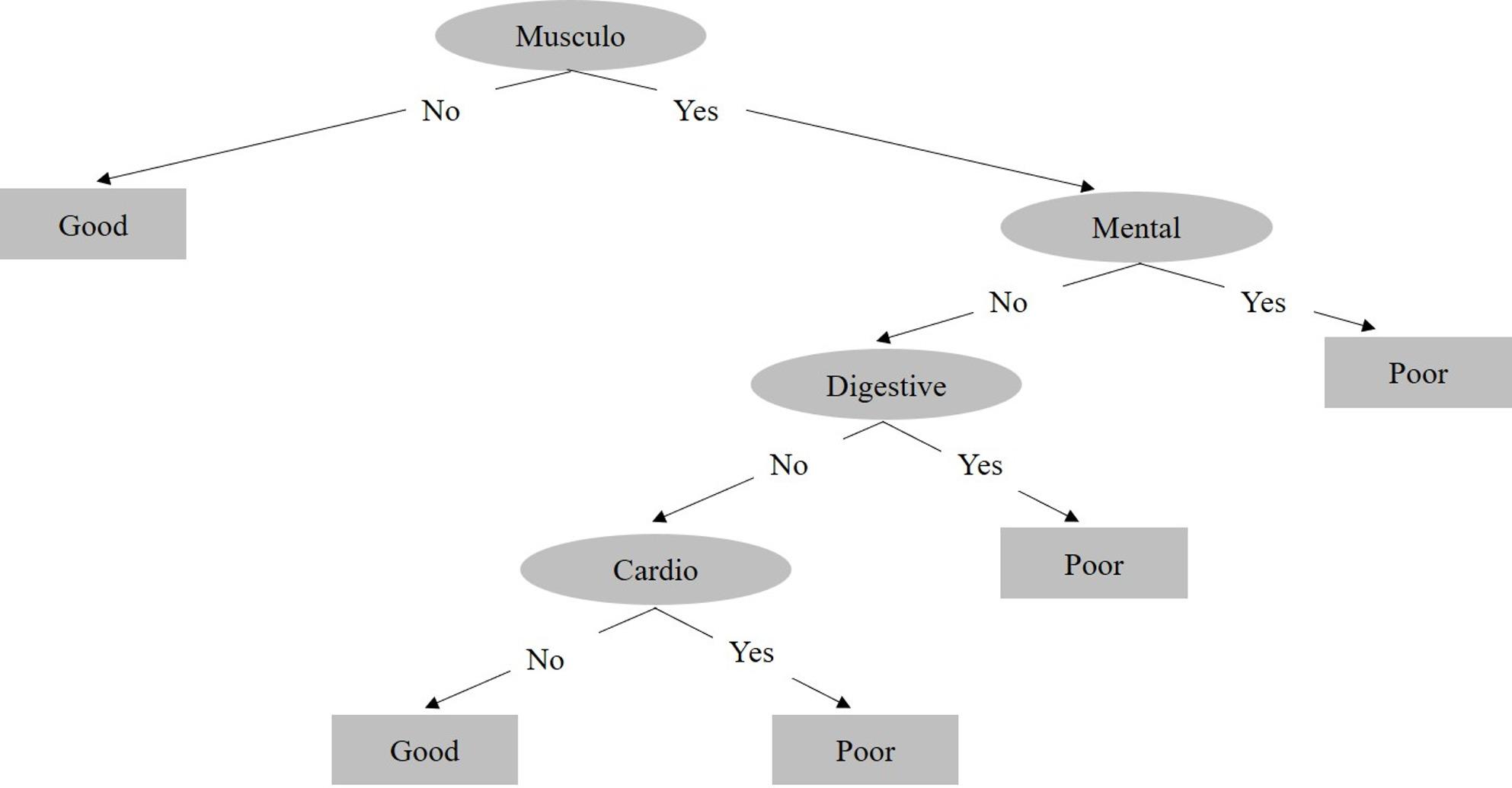
Decision tree for classification of poor self-rated health for men in the intermediate class group

**Fig. 6:**
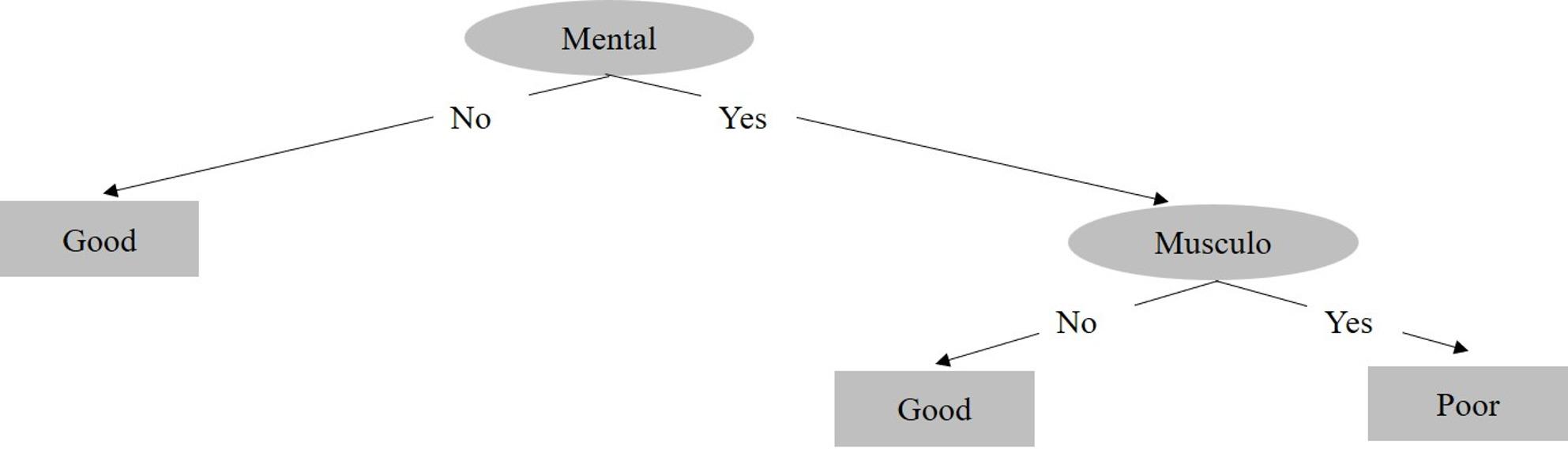
Decision tree for classification of poor self-rated health for men in the professional social class group

The resulting decision trees are shown in Figs. 1, 2, 3, 4 and 5, and 6. For women (Figs. 1, 2 and 3), the socioeconomic gradient shows distinct patterns in the relationships among the health conditions considered. Here, women in the manual class group exhibit greater complexity than those in the professional class. However, this reduced model complexity does not imply lower classification accuracy by the resulting decision tree, as women in the professional class display the highest accuracy compared with real values of SRH (79.4% correctly classified cases, compared to 77.6% among intermediate-class women and 76.5% among manual-class women). These accuracy values for the respective decision trees are closely aligned with those obtained for the Random Forest models across the three intersectional groups (79.5%, 77.7%, and 76.7% for women in professional, intermediate, and manual occupations, respectively), thereby validating the resulting trees.

Among women in the manual group (Fig. 1), the health conditions that contribute to classify their response to the question on SRH are, in this order: mental disorders, musculoskeletal diseases, limitations, respiratory diseases, and the “other” group. The case of mental disorders is noteworthy, as its diagnosis is directly associated with poor SRH. Musculoskeletal diseases emerge at a secondary level owing to their potential to limit the daily lives of affected individuals. Indeed, interactions between these diseases and activity limitations, or with respiratory diseases, lead to a poor SRH. Finally, in the absence of the aforementioned chronic diseases, the interaction between musculoskeletal diseases and the “other” group also results in classification of poor SRH.

In the case of women in the intermediate group (Fig. 2), the role of mental and musculoskeletal diseases is similar to their counterparts in the manual group. The interaction between mental disorders and activity limitations is associated with an estimated poor SRH outcome. The main difference is the contribution of genitourinary chronic diseases, which are associated with a direct estimation of poor health in the absence of mental and musculoskeletal diseases.

Finally. for women in the professional group, (Fig. 3), the only health conditions that are relevant for classifying the current SRH status are musculoskeletal and neurological diseases. Indeed, poor SRH is classified exclusively by the interaction between these two disease groups.

In the case of men (Figs. 4, 5 and 6), distinct patterns across social groups are once again observed concerning the complexity of the decision trees utilised to classify poor SRH. In line with the findings previously described for women, the manual group is associated with greater complexity in the relationships between health indicators required to classify poor SRH. This outcome is confirmed without detriment to the models’ accuracy, as the percentage of correctly classified cases remains highest in the professional group (84.5%) compared to the intermediate (81.2%) and manual (79.7%) groups. Furthermore, the comparison of the accuracy values between the decision trees and the corresponding Random Forests (84.5%, 81.3%, and 79.9% for women in professional, intermediate, and manual occupations, respectively) validates the results obtained.

Unlike the female cohort, musculoskeletal disorders carry greater weight for men in the manual (Fig. 4) and intermediate (Fig. 5) groups. However, these conditions alone do not directly associate with a poor SRH. Among manual workers, they only do so when combined with other morbidities such as genitourinary, cardiovascular, or endocrine disorders. Conversely, in cases where no musculoskeletal disorder is reported, disabilities are shown to be directly associated with the declaration of poor SRH. Finally, the combination of mental and cardiovascular disorders also associates with poor SRH. For the intermediate group, musculoskeletal disorders are consistently present in the classification of poor SRH, appearing in combination with mental, digestive, or cardiovascular disorders.

By contrast, in the group of professional men (Fig. 6), mental disorders assume a prominent role. These conditions, combined with musculoskeletal disorders, constitute the sole pathway associated with poor SRH.

Beyond accuracy, Table 2 presents additional performance metrics for the six decision trees. AUC values ranged from 0.58 (professional men) to 0.75 (manual women), indicating moderate discriminatory ability, with generally higher values observed for manual occupational classes. Sensitivity for good SRH was consistently high across all decision trees (0.79–0.85), whereas sensitivity for poor SRH was substantially lower (0.62–0.77), indicating that the models were more effective at correctly classifying individuals with good SRH. A similar asymmetry was observed for the precision-recall curve (PRC) area: values for good SRH remained stable (0.82–0.85), while PRC area for poor SRH varied widely (0.29–0.61), with the lowest values found in professional groups where poor SRH was least prevalent.

**Table 2:** Predictive performance of the decision trees for SRH by intersectional strata

	Accuracy	AUC	Sensitivity		PRC Area	
			Good	Poor	Good	Poor
Manual women	76.53	0.75	0.80	0.67	0.82	0.61
Intermediate women	77.65	0.71	0.79	0.69	0.83	0.51
Professional women	79.41	0.64	0.81	0.62	0.82	0.40
Manual men	79.71	0.72	0.83	0.64	0.85	0.51
Intermediate men	81.24	0.69	0.84	0.64	0.85	0.46
Professional men	84.47	0.58	0.85	0.77	0.85	0.29

## Discussion

This study assesses the association between of various specific health conditions as activity limitations (ADL and IADL together) and groups of chronic diseases (cardiovascular, respiratory, musculoskeletal, endocrine, mental, neurological, digestive, genitourinary, tumoral, and others), and poor self-rated health (SRH) for the Spanish population aged 30–60, stratified by the intersections of gender and social class. The association was assessed using the classification of poor SRH by J48 decision trees and employing data from the Spanish Health Survey (ESdE) conducted in 2023. This research contributes to a better understanding of the health conditions associated with people’s responses to questions about their general health in a population aged between 30 and 60 years old where health problems are incipient. By applying decision trees, a novel examination of health inequalities is adopted: rather than presenting a unidimensional social gradient in health, it reveals specific configurations in SRH responses that align with the epidemiological profiles of each intersectional stratum. The results demonstrate distinct patterns of health conditions which associate with poor SRH across intersectional strata. When focusing on the effect of social class, it is observed that the less skilled the group, the more complex the structure of the decision tree for classifying poor SRH. Considering that our focus is on the adult age group, this increased complexity may be explained by the fact that this social group experiences a diagnosis of these health conditions at earlier ages, leading to a more rapid accumulation of chronic illnesses, with an increasing relative importance of mental and musculoskeletal diseases [40].

Indeed, in our study, the specific importance of chronic health conditions on poor SRH differs from those observed across older age groups [35]. Among older cohorts, a longer period of coexistence with a disease may consolidate a process of normalization [5], thereby relativizing its influence on the overall health. However, in the younger population, this normalization process has not yet occurred. Consequently, the diagnosis of a chronic illness, in combination with a mental or musculoskeletal disease, or the presence of a limitation in daily activities may directly affect perceptions of one’s own health. Indeed, a state of multimorbidity (i.e. the coexistence of two or more chronic diseases) leads to an estimation of poor SRH. Differences with previously published research on older age groups [10 , 11] confirms that health inequalities emerge through processes unfolding across the life course. Examining these dynamics longitudinally among individuals may enhance our understanding of inequity patterns and help inform policies aimed at mitigating them, thereby overcoming the cross-sectional limitations inherent in our study. The impacts in the intersection of gender and social class may accumulate or change over time, potentially explaining why these results diverge from previous research focused solely on elderly populations.

Our results did not uncover meaningful gender inequalities in the effects of the selected health conditions associated with poor SRH. This confirms previous findings suggesting that, rather than a differing gendered perception of health, what is observed is a meaningfully poorer health profile in women compared with their male counterparts with similar sociodemographic features when older populations are explored [41]. Nevertheless, during youth and adulthood, the shorter timeframe for the accumulation of adverse health situations translates into a more homogeneous health pattern and, therefore, a potentially similar influence on SRH, though this must be confirmed with further research.

From an intersectional lens, the six specific social strata obtained through the mutual reinforcement of gender and social class in this analysis generate decision trees that differ in composition (the health conditions relevant to the classification of poor SRH), shape, and length. The trees are qualitatively distinct across the intersectional strata, consistent with the premise that the effects are not merely additive. This is in line with the intersectional framework’s assumption that individuals across different intersectional strata are groups situated along asymmetric power structures (patriarchy and social class relations under capitalism), and as a consequence, are unequally exposed to social mechanisms (re)producing inequalities (i.e., distanciation, exclusion, hierarchization, and exploitation [42]). As an interpretative hypothesis, higher prevalence of distinct negative health conditions may explain why women and lower social class groups present more complex trees than strata composed of men and higher social class strata, who accumulate privilege.

Notably, a more comprehensive intersectional approach would also incorporate forms of discrimination related to migratory status, ethnicity, or racialization. Although this information is not collected in the Spanish National Health Survey, a significant portion of the population is of migratory origin (as of 2025, 14% of the Spanish population held foreign nationality [43]), alongside a prominent native ethnic group (the Roma population, estimated to represent between 1.5% and 2.1% of the overall Spanish population [44]) and groups racialized through historical processes, including colonization. Furthermore, moving beyond a binary conceptualisation of gender would facilitate a deeper understanding of the internal heterogeneity within this variable. However, the survey questionnaire does not capture this information. At the same time, the binary construction of gender is a structuring feature of capitalist patriarchal societies, with material consequences for the sexual division of labour and the unequal distribution of health-relevant resources and burdens. Accordingly, the binary gender variable available in this survey is analytically meaningful for the purposes of this study.

Beyond the data constraints mentioned above, this research is not without its limitations. The primary limitation is the difference in sample sizes across the analyzed intersectional strata. This factor could influence the structure of the resulting trees, as it is directly related to the pruning criterion. To overcome this limitation and following the principle of parsimony, we aimed to find the optimal solution by balancing maximum classification accuracy with minimal tree complexity, as shown in Additional File 1.

Despite the notably high accuracy levels demonstrated by the various decision trees, a proportion of individuals were misclassified. his issue invariably exerts a more pronounced effect on the less frequent category, poor self-rated health in the context of our study, thereby resulting in a class imbalance that impacts classifier performance. Additionally, from a conceptual standpoint, there other factors such as life satisfaction and happiness [45] that we did not considered have been shown to be associated with SRH, though our selection of health conditions encompasses the primary dimensions of physical health. This further underscores the significance of the subjective dimension of SRH, a topic that warrants dedicated future research. Nevertheless, exploring these subjective determinants was beyond the scope of our research as we opted to focus on objective health conditions to understand the underlying drivers of SRH within our study population.

## Conclusions

This research contributes to the emerging field of study regarding what truly underlies individuals’ general assessment of their health. The process of normalization, which is essential for understanding why some populations with poorer health report more favorable self-evaluations, appears to have a significant effect at older ages. However, for younger and middle-aged cohorts, the process is markedly simplified: a state of multimorbidity, defined as the presence of two or more chronic conditions, is sufficient to show a direct association with poor SRH. While much research remains to be done in this direction, this work contributes to a more detailed understanding of the components of SRH.

Likewise, we consider that the resulting decision trees illustrate the social gradient in health in a novel way that enriches the understanding of health inequalities. Instead of simply showing a linear trend across a one-dimensional variable, these trees reveal that as disadvantages accumulate through various combinations of gender and social class, the combination of relevant diseases do not only become more complex, but also distinct, in specific and unique ways for each group, as the intersectional framework predicts.

## Supplementary Information

Below is the link to the electronic supplementary material.

Supplementary Material 1

## Data Availability

No datasets were generated or analysed during the current study.
